# Success Probability Characterization of Long-Range in Low-Power Wide Area Networks

**DOI:** 10.3390/s20236861

**Published:** 2020-11-30

**Authors:** Yi-Kang Kim, Seung-Yeon Kim

**Affiliations:** Department of Computer Convergence Software, Korea University, Sejong 30019, Korea; kimyikang@korea.ac.kr

**Keywords:** low-power wide area network, long-range, ALOHA, capture model, and chirp spread spectrum

## Abstract

In low-power wide area networks (LPWAN), a considerable number of end devices (EDs) communicate with the gateway in a certain area, whereas for transmitted data, a low data rate and high latency are allowed. Long-range (LoRa), as one of the LPWAN technologies, considers pure ALOHA and chirp spread spectrum (CSS) in the media access control (MAC) and physical (PHY) layers such that it can improve the energy efficiency while mitigating inter-cell interference (ICI). This paper investigates the system throughput of LoRa networks under the assumption that the interferences between EDs for exclusive regions are ignored using CSS. In order to establish an analytical model for the performance of LoRa, we introduce the pure ALOHA capture model, which is the power threshold model. For this model, we assume that the interfering power is proportional to the length of the time overlapped. In addition, we discuss LoRa gain by comparing the total throughput of LoRa with that of non-CSS.

## 1. Introduction

Internet of Things (IoT) affects services such as facility management, smart buildings, connected cities, and manufacturing applications. Low-power wide area networks (LPWAN) appear to be a long-range solution that can respond to the demands of IoT services, which involves the deployment of highly scalable systems employing low-cost edge-devices with low battery consumption [1,2]. Long range (LoRa), Sigfox, LTE-M, and LTE NarrowBand (NB)-IoT are currently the LPWAN technologies with the greatest momentum [3]. In these types of LPWAN technologies, a large number of end devices (EDs) are deployed at an urban scale, and they transmit a relatively small amount of data directly to the base station (BS) or gateway, in a so-called star topology. These systems use the ALOHA-based protocol to take advantage of its low energy consumption for the media access control (MAC) protocol. Moreover, in LPWAN, the EDs use a different physical layer (PHY) to mitigate the effects of interference and noise. In particular, the PHY of LoRa technology is a derivative of the chirp spread spectrum (CSS), while that of Sigfox technology is an ultra-narrow band modulation using binary phase-shift keying.

In this paper, we focus on LoRa proposed by Semtech and promoted by the LoRa Alliance [4], where there are three classes of EDs based on their downlink response time and energy consumption. Class A type EDs offer the best energy-saving performance by waking up only when they have data to transmit using ALOHA. Class B type EDs wake up at periodic intervals to synchronize and exchange data with the gateway. Class C mode has no downlink restrictions and can receive downlink messages any time whenever it is not in a transmitting state. Since class B and class C are extensions to the specification of class A, we consider a class A mode.

While it is well known that the maximum throughput is 1/2e for a pure ALOHA protocol-based system, its throughput is obtained by assuming that a collision occurs when two or more packets arrive at the receiver in an overlapping time period. However, in a practical network, the overlapped packets can be demodulated at the receiver with a certain threshold, which is called the *capture effect*.

In previous work on LoRa, Centenaro et al. [5] and Haxhibeqiri et al. [6] demonstrated that LoRa PHY using CSS can achieve improved performance, in which the MAC protocol’s effect was expected. Magrin et al. [7] and Abeele et al. [8] showed the improved performance of the LoRa system through simulations. In addition, Augustin et al. [9] established some field trials of LoRa EDs and system-level simulations of LoRa MAC procedures to evaluate the throughput of a LoRa network. Reference [10] studied the performance analysis of LoRa modulation, in which the collisions between packets modulated with different SFs were considered. In References [11,12,13,14], the capture effect of LoRa was considered. To improve the capture effect of LoRa, Noreen et al. [11] applied successive interference cancellation scheme, which can allow recovering of the weaker packets. In Reference [12], the authors developed the capture effect model for analyzing the LoRa network, where the propagation loss is considered for the channel model. From a modeling point of view, in this paper, we will consider the capture effect as in References [11,12,13,14]. In contrast to References [11,12,13], we will furthermore consider fading channel model as well as interfering power model according to the overlap time of interfering packet.

As mentioned above, CSS is used as a modulation scheme for LoRa systems that support the six-spreading factor (SF). This can guarantee orthogonality when, for the overlapped packets with different SF, these get a signal to interference and noise ratio (SINR) over a certain isolation threshold [15]. Hence, we only focus on the interference caused by the co-spreading factor interference.

The main contribution of this paper is in analyzing the performance of a pure ALOHA protocol-based LoRa system with the capture effect under the orthogonality of the CSS modulation scheme. In particular, under the infinite population model for a pure ALOHA protocol, we can derive a power threshold-based analytical model for obtaining the upper bound performance for LoRa systems. For the capture effect, we introduce the pure ALOHA capture model, where the interfering power is proportional to the length of time overlapped.

The remainder of this paper is organized as follows. Section 2 introduces the LoRa systems model with CSS scheme. The expression for the success probability is derived in Section 3. The results of the analytical model are presented in Section 4 and conclusions are given in Section 5.

## 2. System Model

**Figure 1:**
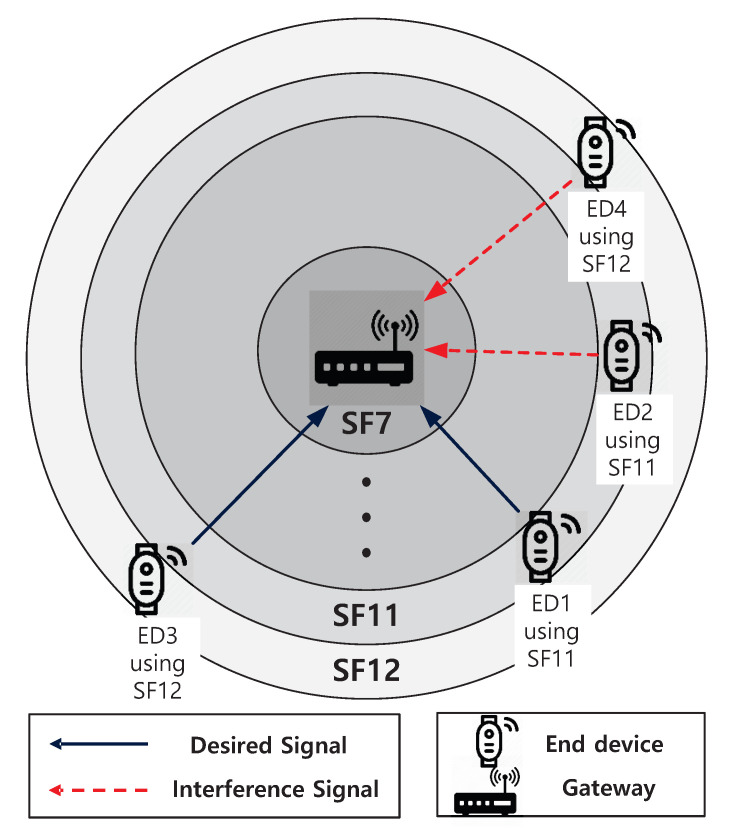
Long range (LoRa) network composed of the end devices (EDs) and the gateway, where the desired signal of ED1 only suffers from the interference signal of ED2 and the desired signal of ED3 only suffers from the interference signal of ED4 by using chirp spread spectrum (CSS).

Suppose an LoRa network composed of EDs and a gateway, where EDs are uniformly deployed over a circular area cell and can communicate with a gateway at the center of the cell. As shown in Figure 1 and Table 1, a cell is divided into six zones which can be referred to as $A_{1}$ to $A_{6}$ according to SF. We assume that the new packet generation process of EDs in a cell follows a Poisson process with a mean request rate of G=λ (packets/s). Then, EDs arriving in a zone *i* generate a packet at an interval, which is exponentially distributed with mean $1/G_{i}=1/\lambda_{i}=1/\left(A_{i}\lambda \right)$, where $A_{i}$ is the fraction of the $i^{th}$ area to the cell. We further assume that one ED can only hold one packet, and that it will not generate a new packet until it has a success or collision for the transmitted packet, where the length of a packet, *T*, is normalized to one. Additionally, we consider the infinite population model where aggregate traffic, that is, the sum of new packets transmitted by non-backlogged EDs and those retransmitted by backlogged ones constitutes a Poisson process.

In our system, successful packet transmission can proceed in two ways: The first one is that in which a tagged packet is to be considered a successful packet transmission when there are no overlapped packets during a period of collision for a tagged packet. The second case is that the signal-to-interference noise ratio (SINR) of a tagged packet received at the gateway is more than a predefined threshold value, where a tagged packet undergoes collisions with other packets. For this case, we assume that, for the asynchronously received packets, the gateway’s receiver attempts to decode the first receiving packet and treats other packets as interfering packets.

Let $P_{s}$ and $P_{cap}$ be the success probabilities of the first and second cases, respectively. For a zone *i*, the throughput of the system with capture is expressed as

(1) $$\begin{array}{c} S_{i}=G_{i}\left(P_{s}^{i}+P_{cap}^{i}\right), \end{array}$$

where $P_{s}^{i}$, as has been well known that the success probability of a pure ALOHA system without the capture effect, is $e^{-2G_{i}}$. Thus, the normalized total throughput is expressed as

(2) $$\begin{array}{c} S=\sum_{i=1}^{6}\frac{S_{i}}{G}=\sum_{i=1}^{6}\frac{G_{i}\left(e^{-2G_{i}}+P_{cap}^{i}\right)}{G}, \end{array}$$

where $P_{cap}^{i}$ denotes the capture effect probability for a zone *i*, which will be derived in the next section.

## 3. Analysis

In this section, we establish the analytic model for the capture effect probability, $P_{cap}$, which is based on the power threshold model. Although, for this model, noise will be ignored in the analysis for the sake of simplicity, we take noise in simulations into account and compare the results of the analysis with the simulation results in Section 4.

**Lemma** **1.** *For T=1, the probability that the first arriving packet will have a collision in region i, $P_{fc}^{i}$, is expressed as*

(3) $$\begin{array}{c} P_{fc}^{i}=e^{-G_{i}}-e^{-2G_{i}}. \end{array}$$

**Proof.**  As mentioned in the system model, we assume that the packet generate process follows a Poisson process with a mean request rate of $G_{i}$ for a region *i*. From this assumption, the probability that a packet is arrived after idle, $P_{f}^{i}$, is expressed as

(4) $$\begin{array}{c} P_{f}^{i}=\frac{G_{i}^{n}}{n!}e^{-G_{i}}{|}_{n=0}=e^{-G_{i}}. \end{array}$$

Note that, for T=1, the success probability without the capture effect is $e^{-2G_{i}}$. Thus, $P_{fc}^{i}=e^{-G_{i}}-e^{-2G_{i}}$. □

In order to establish the power threshold model, we assume that the power of the packet received at the gateway from an ED *j* is as follows

(5) $$\begin{array}{c} P_{j}=P_{t}r_{j}^{-\alpha }h_{j}, \end{array}$$

where $P_{t}$ is the transmission power of a ED and $h_{j}$ indicates the small-scale fading, which is assumed to be exponentially distributed with mean $\varphi_{j}$, that is, Rayleigh fading. $r_{j}^{-\alpha }$ denotes path loss at a distance $r_{j}$ between ED *j* and the gateway, where α denotes the path-loss exponent.

**Figure 2:**
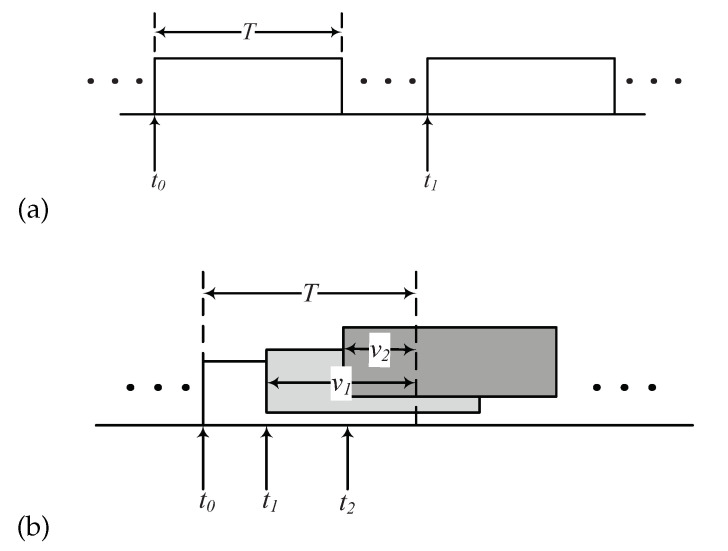
Scenarios of transmitted packets: (**a**) no overlapped packets during a period of collision for a tagged packet, (**b**) a tagged packet undergoes collisions by other packets.

Suppose that the interfering power is proportional to the length of the time overlapped during the transmission time of the target packet, that is, $P_{t}\nu /T$. Then, let us consider that a packet received at $t_{0}$ is corrupted by the other packet transmissions occurring at $t_{1}$ and $t_{2}$ in Figure 2. In such a scenario, the sum of the interference power is $P_{t}\left\{\left(t_{0}+T\right)-t_{1}\right\}/T+P_{t}\left\{\left(t_{0}+T\right)-t_{2}\right\}/T$. Based on this assumption, the SINR of the target packet received at the gateway from the target ED with *m* interfering packets can be expressed as

(6) $$\begin{array}{c} SINR=\frac{P_{t}r_{D}^{-\alpha }h_{D}}{\sum_{m}\nu_{m}P_{t}r_{m}^{-\alpha }h_{m}+\sigma^{2}}, \end{array}$$

where $\sigma^{2}$ denotes additive noise, $r_{D}$ is the distance between the target ED and the gateway, and $r_{m}$ is the distance between the interfering ED and the gateway. Additionally, in (6), $\nu_{m}$ denotes the normalized length of the time interval for interfering packet *m*, which is expressed as

(7) $$\begin{array}{c} \nu_{m}=\frac{\left(t_{D}+T\right)-t_{m}}{T}, \end{array}$$

where $t_{D}$ and $t_{m}$ denote the arrival times of the target packet and interfering packet *m*, respectively.

**Lemma** **2.** *For (7), the mean of $\nu_{m}$,$\bar{\nu }$, is 1/2 and the probability that the SINR of the target ED exceeds some predefined threshold value, $\gamma_{th}$, is*

(8) $$\begin{array}{c} \mathrm{Pr}\left[SINR>\gamma_{th}\right]=\mathrm{Pr}\left[\frac{P_{t}r_{D}^{-\alpha }h_{D}}{\frac{1}{2}\sum_{m}P_{t}r_{m}^{-\alpha }h_{m}+\sigma^{2}}>\gamma_{th}\right]. \end{array}$$

**Proof.**  Note that, although the inter-arrival times of packets are exponentially distributed with mean $1/G_{i}$, the time epochs of the Poisson packet arrivals in a fixed interval *T* are uniformly distributed. Thus, for $t=t_{m}-t_{D}$, it is a uniformly distributed between 0 and *T*, that is, the pdf of *t*, $f_{t}\left(t\right)=1/T$. Thus, the mean of $\nu_{m}$, $\bar{\nu }$, is obtained by

(9) $$\begin{array}{c} \bar{\nu }=\int_{0}^{T}\frac{\left(T-t\right)}{T}f_{t}\left(t\right)dt=\int_{0}^{T}\frac{\left(T-t\right)}{T^{2}}dt=\frac{1}{2}. \end{array}$$

By substituting (9) into (6), the SINR can be expressed as

(10) $$\begin{array}{c} SINR=\frac{P_{t}r_{D}^{-\alpha }h_{D}}{\frac{1}{2}\sum_{m}P_{t}r_{m}^{-\alpha }h_{m}+\sigma^{2}}. \end{array}$$

Therefore, $\mathrm{Pr}[SINR>\gamma_{th}]$ is (8). □

**Lemma** **3.** *When we do not consider the noise, the probability density function (pdf) of the SIR is expressed as*

(11) $$\begin{array}{c} f_{z}\left(z|m\right)=\frac{m\delta }{{\left(\delta z+1\right)}^{m+1}}, \end{array}$$

*where $\delta =1/2{\left(r_{m}/r_{D}\right)}^{-\alpha }$.*

**Proof.**  From a high SIR approximation, (6) can be expressed as

(12) $$\begin{array}{c} z=\frac{h_{D}}{\sum_{m}I_{m}h_{m}+\frac{\sigma^{2}}{P_{t}r_{D}^{-\alpha }}}\approx \frac{h_{D}}{\sum_{m}I_{m}h_{m}}, \end{array}$$

with $I_{m}=\left(r_{m}^{-\alpha }\right)/\left(2r_{D}^{-\alpha }\right)$. For a given $r_{m}$ and $r_{D}$, the upper bound of the received SIR is expressed as

(13) $$\begin{array}{c} z=\frac{1}{\delta }\frac{h_{D}}{\sum_{m}h_{m}}. \end{array}$$

In order to obtain the pdf of *z*, first let $x=h_{D}$ and $y=\sum_{m}h_{m}$. As $h_{D}$ and $h_{m}$ follow exponential distributions, the pdfs of *x* and *y* are $f_{x}\left(x\right)=e^{-x}$ and $f_{y}\left(y\right)=\frac{y^{m-1}}{\Gamma (m)}e^{-y}$, respectively. Note that, for the sum of *m* independent exponential distributions, we used an Erlang-*m*, where Γ(·) is the gamma function. For given *m* interferences, the pdf of γ=x/y is obtained as

(14) $$\begin{array}{cc} f_{\gamma }\left(\gamma |m\right) & =\frac{dF_{\gamma }\left(\gamma |m\right)}{d\gamma }=\frac{d\int_{0}^{\infty }\int_{-\infty }^{y\gamma }f_{x,y}\left(x,y\right)dxdy}{d\gamma }\\ & =\int_{0}^{\infty }yf_{xy}\left(y\gamma ,y\right)dy\\ \; & =\frac{1}{\Gamma (m)}\int_{0}^{\infty }y^{m}e^{-y(\gamma +1)}dy=\frac{m}{{\left(\gamma +1\right)}^{m+1}}, \end{array}$$

where $F_{\gamma }\left(\gamma \right)$ denotes the cumulative distribution function of γ. Next, the pdf of z=γ/δ is obtained as presented in (11), where we have used $f_{z}\left(z|m\right)=\delta f_{\gamma }\left(\delta z|m\right)$. □

**Lemma** **4.** *For a given threshold $\gamma_{th}$ and $G_{i}$, the capture effect probability in a region i can be expressed as*

(15) $$\begin{array}{cc} P_{cap}^{i} & =P_{fc}^{i}\cdot \sum_{m=1}^{\infty }\mathrm{Pr}\left[SIR>\gamma_{th}|m\right]f_{m}\left[m\right]\\ \; & =P_{fc}^{i}\cdot (1-\int_{0}^{\gamma_{th}}\sum_{m=1}^{\infty }f_{z}\left(z|m\right)f_{m}\left[m\right]dz)\\ \; & =P_{fc}^{i}\cdot e^{\frac{-G_{i}\delta \gamma_{th}}{\delta \gamma_{th}+1}}, \end{array}$$

*where $f_{m}\left[m\right]$ denotes the probability that there are m interfering packets, that is, $e^{-G_{i}}G_{i}^{m}/m!$ by the Poisson arrival process.*

**Proof.**  In (15), we evaluate $\int_{-\infty }^{\gamma_{th}}\sum_{m=1}^{\infty }f_{z}\left(z|m\right)f_{m}\left[m\right]dz$ as

$$\begin{array}{cc} \sum_{m=1}^{\infty }\int_{0}^{\gamma_{th}} & f_{z}\left(z|m\right)dzf_{m}\left[m\right]\\ \; & =\sum_{m=1}^{\infty }\left(\int_{0}^{\gamma_{th}}\frac{m\delta }{{\left(\delta z+1\right)}^{m+1}}dz\right)\cdot \left(\frac{e^{-G_{i}}G_{i}^{m}}{m!}\right)\\ \; & \overset{\left(a\right)}{=}\sum_{m=1}^{\infty }\left\{1-{\left(\frac{1}{\delta \gamma_{th}+1}\right)}^{m}\right\}\cdot \left(\frac{e^{-G_{i}}G_{i}^{m}}{m!}\right)\\ \; & \overset{\left(b\right)}{=}1-e^{\frac{-G_{i}\delta \gamma_{th}}{\delta \gamma_{th}+1}}, \end{array}$$

which is reduced to (15). Note that we used $\int \frac{am}{{\left(ax+1\right)}^{m+1}}dx=-{\left(ax+1\right)}^{-m}$ in (a). Further, we used $\sum_{k=1}^{\infty }\frac{u^{k}}{k!}=e^{u}$ in (b). □

## 4. Numerical Studies

In order to characterize LoRa systems, this section presents a normalized total throughput *S* and the packet capture effect probability $P_{cap}$ in (15). First, for the power threshold model, we assume that all EDs have a Rayleigh fading with unit mean, that is, $\varphi_{j}=1$, as shown in Section 2, to see the effect of SF on intra cell interference alone. Therefore, the important parameters that can be varied are $\gamma_{th}$ and *G*. In what follows, we set $P_{t}=17$ (dBm), whereas the noise power spectral density is −174 dBm/Hz for a bandwidth of 125 KHz. Note that, in each figure, the lines denote the analysis results while the marks indicate the simulation results.

**Figure 3:**
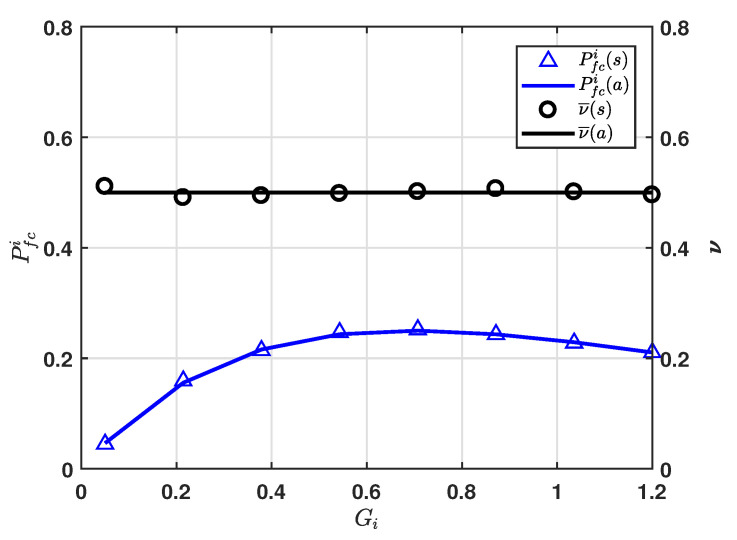
$P_{fc}^{i}$ and $\bar{\nu }$ versus $G_{i}$.

In order to verify Equations (3) and (8), Figure 3 shows the probability that the first packet arrives with collision $P_{fc}^{i}$ and mean length of time interval $\bar{\nu }$ with respect to $G_{i}$. To obtain $G_{i}$ of maximizing $P_{fc}^{i}$, we consider a solution of $\frac{dP_{fc}^{i}}{dG_{i}}=0$. In this way, we can obtain $P_{fc}^{i*}=0.25$ at $G_{i}=\ln2$. $P_{fc}^{i}$ gets the maximum value of 0.25 at approximately 0.7. For $\bar{\nu }$, we demonstrated via simulation that if the time epochs of Poisson packet arrivals at a fixed interval are uniformly distributed, $\bar{\nu }$ remains constant at 0.5.

**Figure 4:**
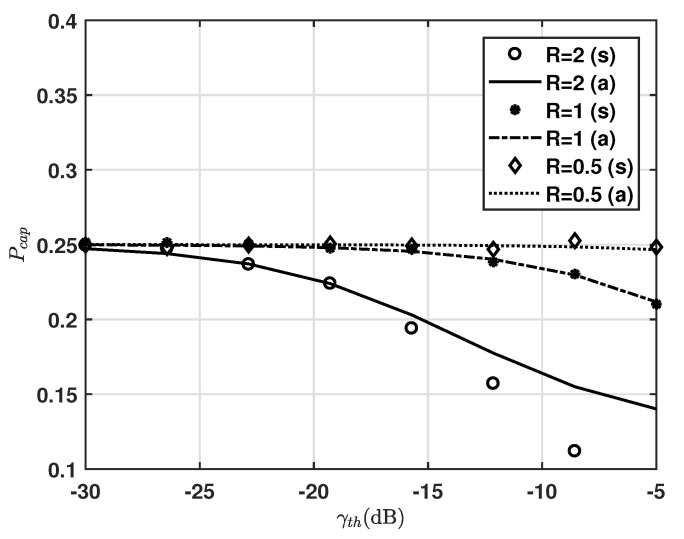
Capture effect probability vs. $\gamma_{th}$ and R

Figure 4 shows the capture effect probability for $-30<\gamma_{th}$(dB) <−5, where $R=r_{D}/r_{m}$ and G=ln2. We can see that, as $\gamma_{th}$ increases, the capture effect diminishes. We can also see that, as *R* decreases, $P_{cap}$ remains constant at 0.25 in varying $\gamma_{th}$. This means that most of the first arriving packets with collision are successfully received for low *R*. It is noticeable that $P_{cap}$ is quite sensitive to the ratio of the distance from target ED to the gateway and from interfering ED to the gateway. The gaps between analysis and simulations are particularly clear for R=2, whereas those for R=0.5 and 1 still show good agreement. Such gaps come from the term whose denominator has an effect of noise, where the simulation result includes the noise effect while Equation (15) does not, while those gaps decrease as *R* decreases. Therefore, we use Equation (15) to obtain the upper bound performance.

**Figure 5:**
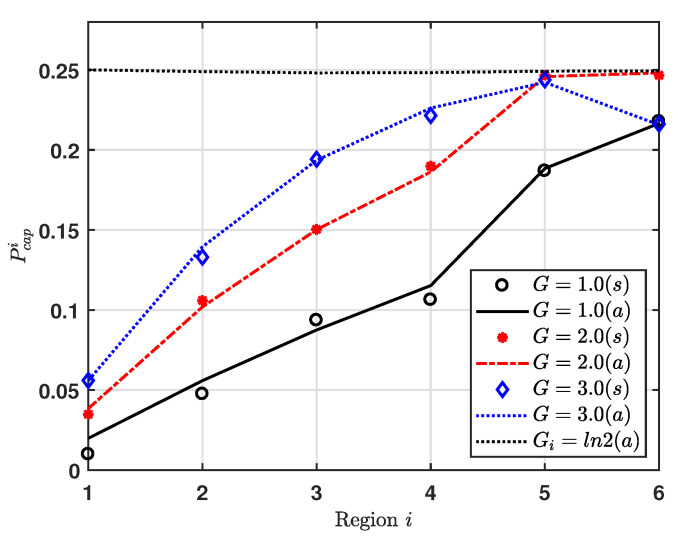
Capture effect probability vs. Region

Figure 5 shows the capture effect probability for region *i*, where EDs are uniformly deployed over a circular area cell for G=1, 2 and 3. Additionally, we consider $G_{i}=\ln2$ for all regions, where that value makes the maximum $P_{fc}^{i}$, as shown in Figure 3. We can see that, for low *G*, the capture effect probability increases as the index of region increases. Further, we can also see that, as *G* increases, $P_{cap}^{i}$ changes. For G=2 and 3, the regions to maximum capture effect probability are regions 6 and 5, respectively. It is noticeable that $P_{cap}^{i}$ is quite sensitive to not only *G* but also the area of region. For example, $G_{6}$ and $G_{5}$ obtain about ln2 at G=2 and G=3, respectively.

**Figure 6:**
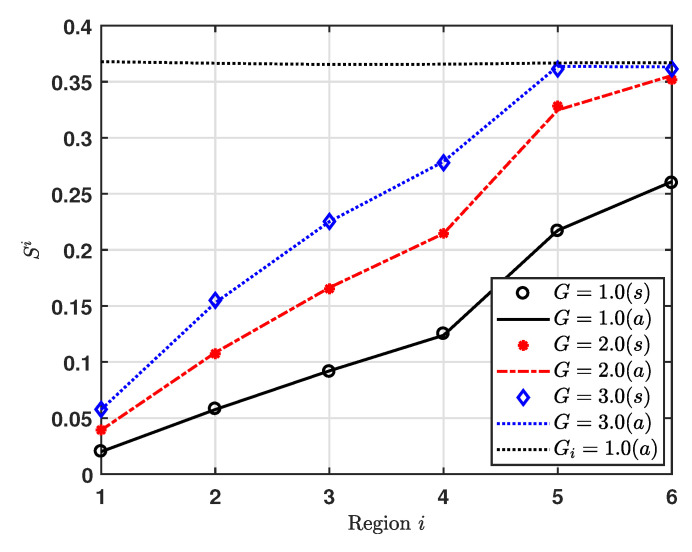
Normalized throughput vs. Region.

Figure 6 shows the normalized throughput for region *i*, at G=1, 2, and 3. Additionally, we consider $G_{i}=1$ for all regions, where to obtain $G_{i}$ of maximizing $S_{i}$ on the upper bound condition, we consider a solution of $\frac{dS_{i}}{dG_{i}}=0$. Then, we get $S_{i}^{*}=0.3679$ at $G_{i}=1$. As expected from the capture effect probability result, the normalized throughput increases as the index of region increases for G=1 and 2. For G=3, the region 5 gets the maximum normalized throughput.

**Figure 7:**
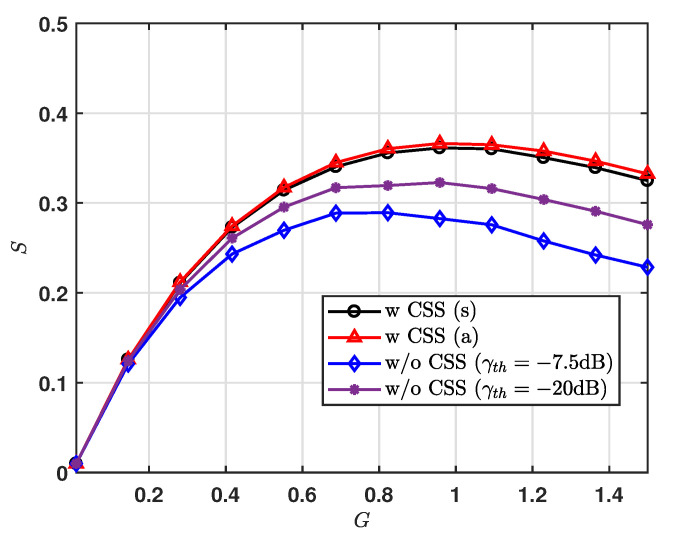
Normalized total throughput vs. G.

In Figure 7, we present the normalized total throughput, *S*, for varying *G*. For the system without the CSS modulation scheme, we set $\gamma_{th}$(dB)=−7.5 and −20, and obtain the results via simulation only, where the noise effect is considered for simulation results. We can see that, for the result of CSS scheme, the gaps between upper bound analysis and simulations are closed. It is noticeable that most of the first arriving packets with collisions are successfully received. Further, we can also see that the CSS scheme can achieve the increased normalized throughput for a given *G*. As expected from the result of the normalized throughput for region, we observe that the LoRa system using the CSS scheme can achieve maximum *S* at G=1.0.

## 5. Conclusions

LPWANs would be an enabler of the internet of things (IoT), where EDs are randomly deployed over a wide coverage area. For those EDs, some vendors consider pure ALOHA as an access system. In this paper, we have introduced an analytic model with which to evaluate the performance of the LoRa system as one of the LPWAN. Specifically, we proposed a capture effect model for a pure ALOHA, where interfering powers with overlapped time lengths are related to each other. We analyzed its performance in terms of normalized throughput by applying CSS. Our analytical results show mostly good agreement with the simulations. The results also indicate that the performance mainly depends on the location of EDs (the region of EDs) and offered load. Since we have assumed that all the EDs use the same transmission power, our results are conservative. Thus, as a future work, for more improved performance of system, it is needed to see the capture effect probability of LoRa system including the power control according to distance between ED and gateway.

## Figures and Tables

**Table 1:** LoRa Spreading factor versus Range and demodulator signal-to-interference noise ratio (SINR).

Region	Spreading Factor	Range	LoRa Demodulator SINR
1	SF7	2 km	−7.5 dB
2	SF8	4 km	−10 dB
3	SF9	6 km	−12.5 dB
4	SF10	8 km	−15 dB
5	SF11	11 km	−17.5 dB
6	SF12	14 km	−20 dB
